# Neurogenesis suggests independent evolution of opercula in serpulid polychaetes

**DOI:** 10.1186/1471-2148-9-270

**Published:** 2009-11-23

**Authors:** Nora Brinkmann, Andreas Wanninger

**Affiliations:** 1 Department of Biology, Research Group for Comparative Zoology, University of Copenhagen, Universitetsparken 15, DK-2100 Copenhagen, Denmark

## Abstract

**Background:**

The internal phylogenetic relationships of Annelida, one of the key lophotrochozoan lineages, are still heavily debated. Recent molecular analyses suggest that morphologically distinct groups, such as the polychaetes, are paraphyletic assemblages, thus questioning the homology of a number of polychaete morphological characters. Serpulid polychaetes are typically recognized by having fused anterior ends bearing a tentacular crown and an operculum. The latter is commonly viewed as a modified tentacle (= radiole) and is often used as an important diagnostic character in serpulid systematics.

**Results:**

By reconstructing the developmental neuroanatomy of the serpulid polychaete *Spirorbis *cf. *spirorbis *(Spirorbinae), we found striking differences in the overall neural architecture, the innervation pattern, and the ontogenetic establishment of the nervous supply of the operculum and the radioles in this species. Accordingly, the spirorbin operculum might not be homologous to the radioles or to the opercula of other serpulid taxa such as *Serpula *and *Pomatoceros *and is thus probably not a part of the tentacular crown.

**Conclusion:**

We demonstrate that common morphological traits such as the prostomial appendages may have evolved independently in respective serpulid sublineages and therefore require reassessment before being used in phylogenetic analyses. Our findings corroborate recent molecular studies that argue for a revision of serpulid systematics. In addition, our data on *Spirorbis *neurogenesis provide a novel set of characters that highlight the developmental plasticity of the segmented annelid nervous system.

## Background

A number of classical and recent studies have shown that structural similarities among organisms are not necessarily based on homologous characters, but may instead be the result of convergent (analogous) evolution. Thereby, these matching phenotypes have emerged independently in distantly related organisms [1-6]. Accordingly, if misinterpreted as sharing common ancestry, convergently evolved characters might lead to false conclusions in phylogenetic reconstructions.

In the age of genomics, convergent evolution is mostly inferred based on existing phylogenies (i.e., *after *the actual analysis) and is often used to explain incongruencies between the phylogenetic signal obtained from molecular *versus *morphological datasets. In this respect, neuronal innervation patterns of organ systems may be used as an independent test of homology *prior *to phylogenetic analysis, as illustrated by classical studies on the structure of the polychaete nervous system [7-9]. Along these lines, it seems reasonable to assume that investigations of neuronal innervation patterns and neurogenesis pathways might also aid in resolving long standing questions regarding the internal phylogenetic relationships of Annelida, one major lophotrochozoan lineage. In particular, the basal branches of the annelid tree remain unresolved, despite the additional use of large-scale molecular analyses [10-19]. Current interest has therefore partly shifted to lower taxonomic levels, taking a "top down" approach to reconstruct annelid phylogeny.

**Figure 1:**
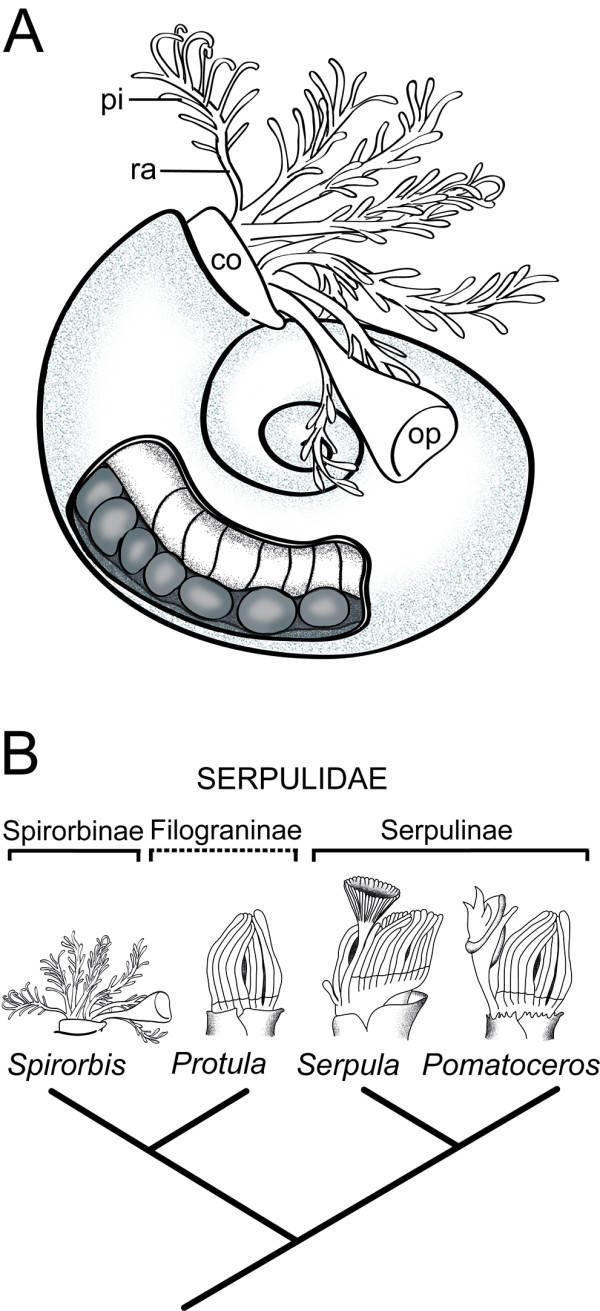
**Serpulid morphology and phylogeny**. (A) Schematic drawing of *Spirorbis *cf. *spirorbis *with the collar (co), the branchial crown consisting of radioles (ra) and pinnules (pi), and the operculum (op) protruding from the coiled shell. The latter is dissected open in the region of the anterior abdomen to unveil the membraneous tube surrounding the embryos. (B) Current hypothesis of serpulid phylogeny based on molecular analyses, with radiole and opercular morphology illustrated for each clade. Sketches redrawn after Kupriyanova et al. [21]. The Serpulidae consist of the Spirorbinae including *Spirorbis *and three new taxonomic entities referred to as the *Protula*-, *Serpula*-, and the *Pomatoceros*-clade. Thereby, some species of the former Serpulinae cluster within the *Protula*-clade that comprises all species of the former Filograninae.

One widely distributed and abundant group of polychaete fan worms, the Spirorbinae, is characterized by a crown of tentacles, commonly referred to as "radioles", and a conspicuous calcareous tube that can be closed with an operculum (Figure 1A). All spirorbins described to date brood their embryos either in their coiled tube or in the operculum, unlike most species of the other two traditional subtaxa of the serpulid tube worms, the Serpulinae and Filograninae [20,21]. A recent cladistic analysis of the relationships among spirorbid subfamilies [22] suggests thereby that the opercular brooders occupy the most derived position on the spirorbin tree. Ever since Müller [23] published his observations on the development of *Serpula *(Serpulinae), the operculum has been viewed as a modified tentacle (radiole) of the branchial crown, and it is often used as an important diagnostic character in serpulid systematics [21,24]. Accordingly, some authors regard spirorbins as a separate taxonomic entity, based on their unique morphology and development [e.g., [25-29]]. However, recent studies of serpulid phylogeny using morphological and molecular data recognize Spirorbinae as one of altogether four monophyletic clades within Serpulidae (Figure 1B). Moreover, they appear to be the sister group to a mixed clade of serpulin and filogranin taxa [21,24], although the statistical analyses could not significantly reject the hypothesis that Spirorbinae are instead the basal-most taxon of the Serpulidae [24]. In addition, the current knowledge and status of the taxonomy of the individual serpulid genera, except the ones of the Spirorbinae, has been reviewed only lately [30].

In order to test the proposed homology - and thus the potential usefulness for phylogenetic analyses - of one of the most crucial morphological characters in the serpulid bodyplan, the operculum, and to assess the hypothesis that all spirorbin opercula evolved from a cephalic radiole, we investigated the ontogeny of the innervation pattern of the prostomial appendages of the spirorbin *Spirorbis *cf. *spirorbis*. In addition, we provide a new set of characters for a broad comparison of neurogenesis in polychaete species.

## Results

### General development and FMRFamidergic immunoreactivity

**Figure 2:**
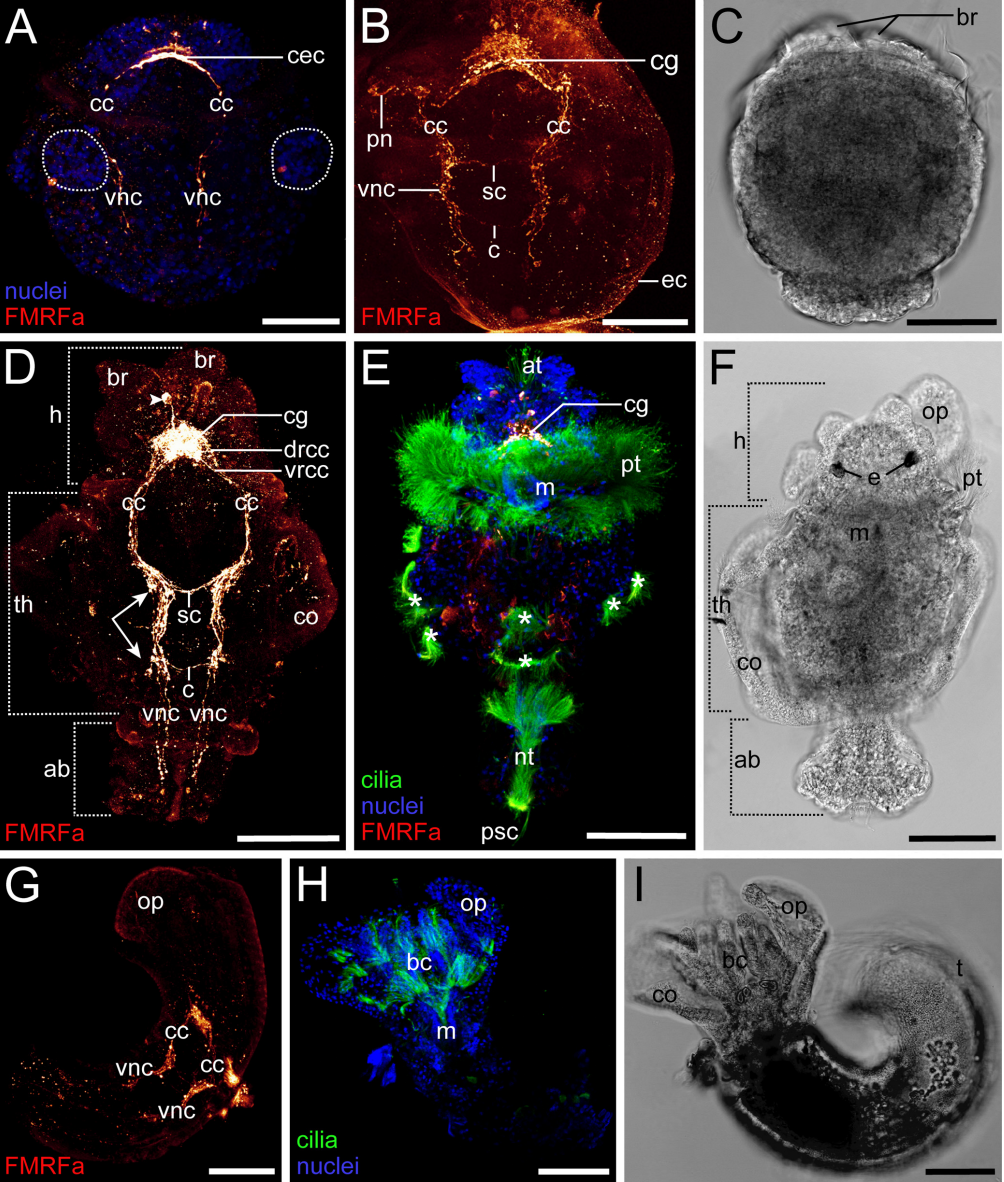
**FMRFamidergic neurogenesis in *Spirorbis*cf. *spirorbis***. Light micrographs and confocal micrographs showing FMRFamide immunoreactivity (red), nuclei (blue), and cilia (green), anterior faces upwards. Scale bars: 50 μm (A-C); 70 μm (D-I). (A-C) Encapsulated larva, dorsal view. (A) Early anlage of the cerebral commissures (cec), the circumesophageal connectives (cc), and the ventral nerve cords (vnc), together with two lateral cell clusters of the collar rudiments (dashed circles). (B) Still within the egg capsule (ec), the cerebral ganglion (cg), the subesophageal commissure (sc), the neuronal innervation of the prototroch (pn), and a commissure (c) are formed in the early trochophore larva. (C) Anteriorly, the paired branchial rudiments (br) are distinguishable. (D-F) Free-swimming larva, ventral view. (D) Three body regions can be recognized: head (h), thorax (th) with collar (co), and abdomen (ab). The ventral nerve cords (vnc) consist of multiple axons in the collar region (arrows), but only of two axons in the posterior body. Anteriorly, one transitional perikaryon (arrowhead) appears and the circumesophageal connectives split into a dorsal (drcc) and a ventral root (vrcc). (E) The larva possesses an apical tuft (at), a prototroch (pt), a mouth opening (m), a neurotroch (nt), several posterior sensory cilia (psc), and various ciliated fields (asterisks). (F) The operculum (op) starts to form and two pigmented eyes (e) are present. (G-I) Settled juvenile. Dorsal view in G, ventral view of the same specimen in H-I. (G) The FMRFamide expression is restricted to head and thorax. (H) Ciliated bands are reduced to the branchial crown (bc). (I) The direction of the coiled tube (t) has been determined.

**Figure 3:**
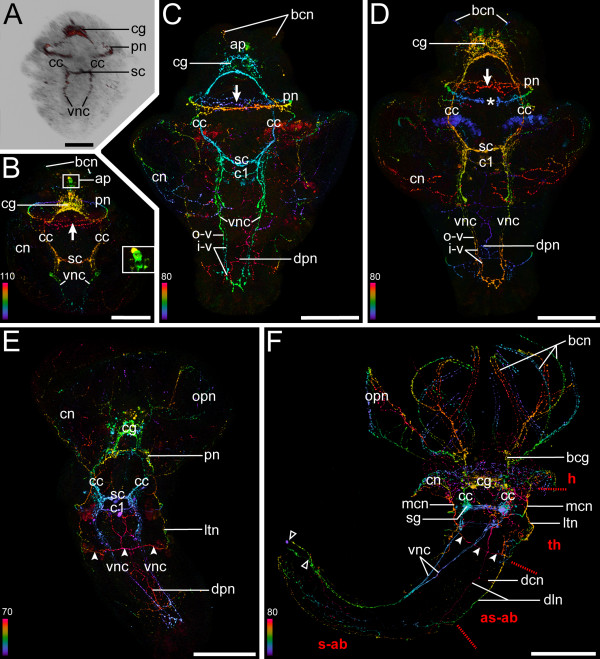
**Serotonergic neurogenesis in *Spirorbis *cf. *spirorbis***. All confocal images are depth-coded except for (A) which is an overlay of a light and the respective confocal image. Anterior faces upwards, color bars indicate thickness of specimens in μm. Scale bars: 50 μm (A); 60 μm (B-D); 75 μm (E-F). Dorsal view in (A, C, E-F) and ventral view in (B and D). (A-B) Encapsulated larva. (A) The cerebral ganglion (cg), the circumesophageal connectives (cc), the ventral nerve cords (vnc), the prototroch nerve ring (pn), and the subesophageal commissure (sc) have started to form. (B) Note the neuronal network of the collar (cn), the cross-linked fibers of the prototroch nerve ring on the ventral side (arrow), the apical perikarya (ap) (magnified in inset), and the neurons of the branchial crown (bcn). (C-D) Free-swimming larva. (C) One additional commisure (c1) and dorsal peripheral neurons (dpn) are present. The ventral nerve cords consist of inner (i-v) and outer (o-v) neurites. (D) Note the dorsal gap in the prototroch nerve ring (asterisk). (E) Settled larva with neurons innervating the operculum (opn), lateral longitudinal nerves (ltn), and a transversal thorax nerve (arrowheads). (F) Settled juvenile with head (h), thorax (th), asetigerous-abdomen (as-ab), and setigerous-abdomen (s-ab). Subesophageal ganglia (sg) and branchial crown ganglia (bcg) develop, together with one main collar neuron (mcn) on either side. The ventral nerve cords (vnc) are not fused at the posterior-most end (open arrowheads). Paired dorsal longitudinal neurons (dln) with a commissural neuron (dcn) appear.

**Figure 4:**
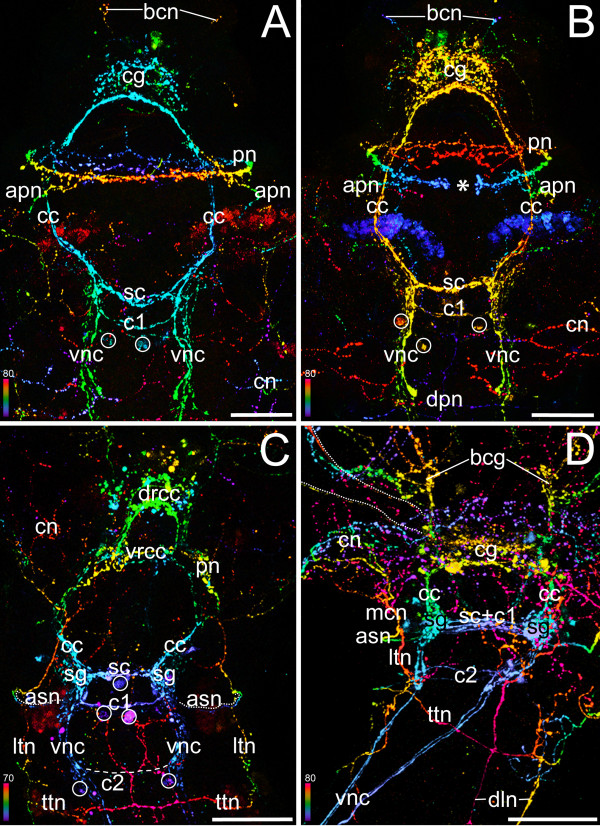
**Serotonin immunoreactivity in the anterior regionof larval and juvenile *Spirorbis *cf. *spirorbis***. Confocal micrographs showing details of Figure 3C-F arranged in the same order. Anterior faces upwards, color bars indicate thickness of specimens in μm. Scale bars: 30 μm (A-B); 40 μm (C-D). (A-B) Free-swimming larva. (A) Dorsal view. Note the growth cones of the branchial crown neurites (bcn) and the collar neurons (cn). The cerebral ganglion (cg) is connected via the circumesophageal connectives (cc) to the ventral nerve cords (vnc) with associated perikarya (encircled) and the subesophageal commissure (sc). The latter is posteriorly followed by one additional commissure (c1). The prototroch nerve ring (pn) is linked to accessory prototroch neurons (apn) on the dorsal side. (B) Ventral view. The prototroch nerve ring (pn) has a dorsal gap (asterisk). (C) Settled larva, dorsal view. Anteriorly, the circumesophageal connectives (cc) split into a dorsal (drcc) and a ventral root (vrcc). The subesophageal ganglion (sg) starts to form. The peripheral nervous system in the thorax region includes an anterior segmental neuron (asn, dotted line), a lateral longitudinal neuron (ltn), and a transversal thorax neuron (ttn). The dashed line marks the very weak signal of the second commissure (c2). (D) Settled juvenile, dorsal view. The two dorsal longitudinal neurons (dln) and the left main collar neuron (mcn) are visible. The ventral commissures (c1 and c2) have been shifted forwards because of the longitudinal compression of the anterior body. The weak signal of the two nerve fibers innervating the operculum is outlined by dashed lines.

**Figure 5:**
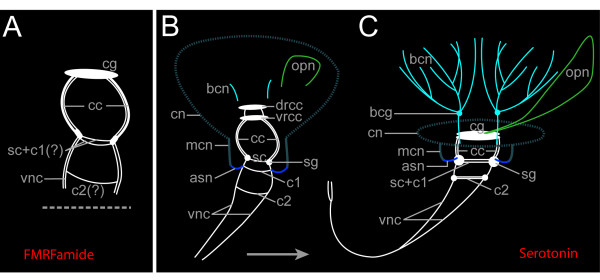
**Central nervous system (CNS) and anteriorperipheral nervous system in *Spirorbis *cf. *spirorbis***. (A) shows the FMRFamidergic and (B) and (C) the serotonergic ganglia and nerves. Apical is to the top. The CNS structures are depicted in white. (A) Settled larva. The neuropeptide FMRFamide is restricted to the CNS structures such as the cerebral ganglion (cg), the circumesophageal connectives (cc), the ventral nerve cords (vnc), the subesophageal commissure, most likely with adjacent first commissure [sc + c1 (?)], and an additional ventral commissure [most probably the second commissure, c2(?)]. These commissures most probably correspond to the ones shown in (B) and (C). (B) Settled larva. The dorsal root (drcc) and the ventral root of the circumesophageal connectives (vrcc) appear as separate entities. The two strands of the ventral nerve cord (vnc) are linked to each other via a subesophageal commissure (sc) in the position of the subesophageal ganglia (sg), and via two additional commissures (c1 and c2). The branchial crown neurons (bcn) and the neurons of the operculum (opn) start to form. They are enclosed in the network of the collar neurons (cn) emanating from the main collar neuron (mcn) on each side of the body. The latter are connected via the anterior segmental neurons (asn) to the CNS. (C) Settled juvenile. Note the condensation of the CNS: the strands of the ventral nerve cords (vnc) are fused in the posterior body, the commissures c1 and c2 are shifted forward, and the circumesophageal connectives (cc) are shortened. bcg, branchial crown ganglia.

Each embryo of *Spirorbis *cf. *spirorbis *is encased in an egg capsule and several dozens of embryos are surrounded by a common membrane aligned along the trunk of the mother individual within the calcareous tube (Figure 1A). Therein, the embryos develop into three-segmented lecithotrophic larvae. These larvae exhibit a short planktonic phase after hatching, settle after a few hours, metamorphose into asymmetric juveniles, and eventually secrete a coiled tube (Figure 2C, F, I). The anlage of the collar, which surrounds the branchial crown of the adult, is already visible in the encapsulated larva (Figure 2A). At the same time, the first FMRFamidergic signals appear in structures of the precursors of the adult central nervous system. The cerebral commissures show an intense staining and are in direct contact with the rudiments of the adult circumesophageal connectives whose signal is faint in the region of the prototroch nerve ring. The ventral nerve cords consist of paired FMRFamidergic axons (Figure 2A). Slightly later, the cerebral commissures fuse, with additional fibers forming the neuropil of the cerebral ganglion (Figure 2B). At the junction of the circumesophageal connectives and the paired ventral nerve cords, a subesophageal commissure interconnects both ventral nerve cords (Figure 2B). The latter vary along the longitudinal body axis with respect to the number of axons they comprise. Anteriorly, the number of neurons with an FMRFamidergic signal increases, whereas only two axons are present in the posterior region. A second commissure appears exactly in the region where this change of axon number occurs (Figure 2B). Prior to hatching, the branchial rudiments and the first setae are already present (Figure 2C). Eventually, after the release of the free-swimming larva, three body regions are recognized: head, thorax with collar, and abdomen (Figure 2D, F). Anteriorly, the circumesophageal connectives split into a dorsal and a ventral root. Both traverse the cerebral ganglion that exhibits a very strong FMRFamidergic signal (Figure 2D). At this stage, the larva possesses various ciliated regions, such as the prototroch, the neurotroch, the apical tuft, and several posterior sensory cilia (Figure 2E). One transitional FMRFamidergic perikaryon is located posterior to the apical cilia (Figure 2D). Apart from that, none of the other ciliated structures shows FMRFamidergic innervation (Figure 2E). The same applies to the operculum that develops dorsally on the left side, in close proximity to the rudiments of the branchial crown (Figure 2F, G). In the following, the larva settles, attaches its ventral side temporarily to the substrate, and finally turns by 180°, resulting in the dorsal side of the thorax facing the substrate. This rotation is not complete in the posterior body region, where the larval abdomen is only twisted by a mere 90°. Therefore, the former ventral and dorsal parts are now located in a (morphological) lateral position. In sharp contrast to the broad distribution of serotonin (Figure 3; Figure 4; Figure 5) in post-settled juveniles, the FMRFamidergic expression is almost exclusively restricted to the head and thorax (Figure 2G; Figure 5A). All larval ciliary bands have been shed, leaving the developing branchial crown the only external ciliated body region. The sinistral direction of the coiled tube has already been determined at this stage (Figure 2H, I).

### Development of the serotonergic nervous system

The neurotransmitter serotonin is present in *Spirorbis *cf. *spirorbis *from the early trochophore stage onwards (Figure 3; Figure 4). The first serotonergic signals appear more or less simultaneously with the FMRFamidergic immunoreactivity. Initially, serotonin is found in the early anlagen of the adult central nervous system, such as the cerebral ganglion, the circumesophageal connectives, the ventral nerve cords, and the subesophageal commissure (Figure 3A). In addition, serotonin is distributed in a nerve ring underlying the larval prototroch. This serotonergic innervation of the ciliated prototroch changes considerably over time (Figure 3; Figure 4). The primary prototroch nerve ring expands on its ventral side shortly before hatching and forms a network of cross-linked serotonergic fibers (Figure 3B, C, D). Moreover, two clusters of accessory prototroch neurons develop dorsolaterally in the free-swimming larva (Figure 4A, B), and a broad gap appears in the serotonergic prototroch innervation (Figure 3D; Figure 4B). After settlement, the neurons of the prototroch are reduced to a semi-circle on the dorsal side and degenerate completely during metamorphosis (Figure 3E, F).

The apical organ of free-swimming *Spirorbis *larvae initially comprises four serotonergic flask-shaped perikarya (Figure 3B). Three of these cells lie in one plane, with the fourth cell body being positioned medially above them. All apical perikarya possess projections that seem to be connected to the rudiment of the adult cerebral ganglion. Interestingly, the number and position of these apical serotonergic somata varies over time as well as between individuals with a maximum of seven being present in specimens competent to settle.

The cerebral ganglion is one of the first adult features that are established during neurogenesis. It is divided into two distinct serotonergic hemispheres (left and right) in the encapsulated and free-swimming larval stages. Posterior to the two domains, the cephalic commissures appear as one strand which is directly connected to the circumesophageal connectives (Figure 4B). After settlement, the latter split anteriorly into the so-called dorsal and ventral roots. Each root differentiates into a dorsal and a ventral commissure. Accordingly, four main commissures, all derived from the circumesophageal connectives, traverse the cerebral ganglion (Figure 4C). The cerebral commissures come to lie in close proximity to the subesophageal commissure, because the circumesophageal connectives are relatively reduced in length in juvenile specimens (Figure 3F; Figure 4D; Figure 5C).

Two thin axons represent the first rudiments of the developing serotonergic ventral nerve cords while the larva is still within the egg capsule (Figure 3A). In later larval stages, the signal of the ventral nerve cords is discontinuous in the second segment. Anteriorly, two axons are present on each side, followed posteriorly by a discrete perikaryon with one long axon pointing medially towards the posterior end (Figure 3B). The ventral nerve cords consist of multiple fibers in the collar region during the free-swimming larval stage and are interconnected by an additional commissure just posterior to the already established subesophageal commissure (Figure 3C, D, E; Figure 4A, B, C). In the abdomen, the ventral nerve cords show a different organization. Thereby, it appears that the axons of the inner ventral nerve cords (i-v) derive from the posterior ectoderm, whereas the ones of the outer part (o-v) are extensions from the anterior thorax region (Figure 3C, D). After settlement, a second commissure is formed in the region where the most anterior neurites of the ventral cords terminate (Figure 4C). The architecture of the ventral nervous system changes significantly with the onset of metamorphosis, eventually resulting in the ventral nerve cords being located on the left side in early juvenile specimens due to the rotated body axis (Figure 3F; Figure 5B, C). Another alteration relates to the shift of the ventral commissures in an anterior direction, resulting in the first ventral commissure merging into the newly formed subesophageal ganglion (Figure 4D; Figure 5C). Furthermore, the two strands of the ventral nerve cords fuse in the segmented setigerous abdomen, except for the posterior-most end (Figure 3F; Figure 5C). Several serotonergic perikarya are associated with the ventral nerve cords from the free-swimming larval stage onwards (Figure 4A, B, C). The number and position of these cells, which are arranged in three clusters, is not consistent among individuals of the same stage. On the opposite side of the abdomen, dorsal longitudinal neurons are present. Interestingly, these two widely separated strands of the peripheral nervous system have a commissural neuron (Figure 3F).

The development of the peripheral nervous system starts with a network of collar neurons in the encapsulated larva (Figure 3B-F). In a second step, dorsal peripheral neurons that innervate the abdomen appear during the planktonic larval phase (Figure 3C, D, E). After settlement, these are directly connected to a transversal thorax nerve which crosses the dorsal body region (Figure 3E; Figure 4C). One lateral longitudinal nerve runs along each side of the thorax from the transversal thorax nerve to the main collar nerve (Figure 3E; Figure 4C). Here, anterior segmental neurons branch off. They are associated with the first ventral commissure of the paired nerve cords (Figure 4C; Figure 5B). These anterior segmental neurons are connected to the subesophageal ganglion after metamorphosis, due to a forward migration of the first ventral commissure (Figure 4D; Figure 5C). Moreover, the lateral longitudinal nerves are shortened compared to the pre-metamorphic condition, and the transversal thorax nerve is connected to the ventral nerve cords at the position of the second ventral commissure (Figure 4D).

### Innervation of the branchial crown and the operculum

**Figure 6:**
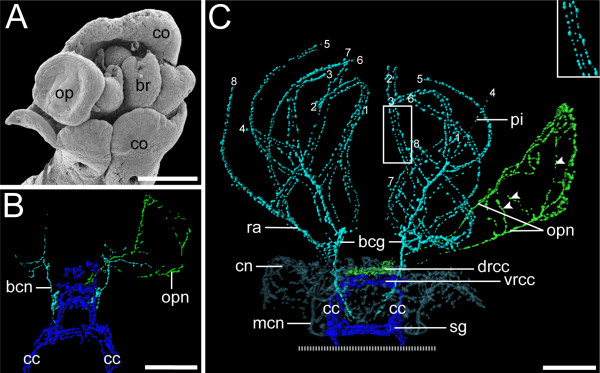
**Development of operculum and radiole innervationin *Spirorbis *cf. *spirorbis***. Scanning electron micrograph (A) and 3D reconstructions of serotonin immunoreactivity in corresponding body regions (B, C). Scale bars: 40 μm. Apical is to the top. (A) Dorsal view with the collar (co) enclosing the rudiments of the branchial crown (br) and the operculum (op). (B) Dorsal view, recently settled larva. Neurons of the branchial crown (bcn; in *turquoise*), the operculum (opn; in *green*), and the circumesophageal connectives (cc; in *blue*) are visible. Note that development of the neuronal innervation of the operculum is ahead of neurogenesis of the radioli. (C) Ventral view, settled juvenile. The neuronal innervation of the branchial crown (*turquoise*) shows no connection to the one of the operculum (*green*). The latter comprises a loop-like opercular nerve (opn) and neurons that irregularly interconnect its two branches (arrowheads). The filaments of the branchial crown, radioli (ra), and pinnulae (pi) are each innervated by three nerves (inset) that branch off from the two branchial crown ganglia (bcg). A clear designation of radioli and pinnulae is partly hindered by their similar appearance at this developmental stage. Therefore, the distal tips of the branchial filaments are consecutively numbered (1-8), indicating their symmetrical order. Note the concentration of the central nervous system (*blue*) with shortened circumesophageal connectives (cc) and newly formed subesophageal ganglia (sg). The neuronal network of the collar (cn) with the two main collar neurons (mcn) is shown in semi-transparent *blue-grey*.

In adult *Spirorbis *specimens, the branchial crown consists of two branches, the left one carrying four and the right one bearing five tentacles (radioli) (Figure 1A). Terminal filaments, the so-called pinnules, branch off from each radiole. The opercular stalk emerges next to the radioli on the left side (Figure 1A; Figure 6A). While serotonin is widely distributed in the whole nervous system of the branchial crown and the operculum, we did not detect FMRFamide in these structures.

The earliest serotonergic signals from the two neuronal growth cones of the branchial crown are present in the encapsulated larva, and additional branchial neurons appear in the free-swimming larval stage (Figure 3B, C, D; Figure 4A, B; Figure 6B). Several axons outline the contour of the operculum shortly after settlement (Figure 3E; Figure 6B). At the same time, the two main nerves of the tentacular crown are already established and split dichotomously at their anterior end (Figure 6B). During metamorphosis, two branchial crown ganglia develop at the base of the tentacles (Figure 6C). From there, neurons branch off and project into the radioli. Exactly the same number of branchial filaments arises on either side. At this point of development, it is not possible to distinguish pinnules from radioles. Eventually, three distinct nerves innervate each individual branchial filament. Accordingly, all radioli and pinnules have one median nerve and two lateral branchial nerves (Figure 6C). The latter are located in a different focal plane than the internal nerve. By contrast, the opercular peduncle and lid are only lined by a single, loop-like opercular nerve. The tissue in the middle of the peduncle is innervated by axons that are irregularly linked to the opercular nerve (Figure 6C). The neurons at the base of the peduncle emerge from the dorsal cerebral commissures, whereas the branchial crown nerves emerge from a more posterior region (Figure 6C). Moreover, the branchial crown nerves reach almost to the subesophageal ganglia. Accordingly, the neuronal innervation of the branchial crown and the operculum, respectively, arises from two different roots which emerge from two distinct regions of the cerebral ganglion in post-metamorphic *Spirorbis *specimens.

## Discussion

### Comparative aspects of polychaete neurogenesis

Despite a significant increase of data on lophotrochozoan neurogenesis and larval neuroanatomy [31-50] and the crucial role of polychaetes for evolutionary inferences, detailed studies on the ontogeny of their nervous system employing immunocytochemical methods are as of yet only available for four species [31,40,43,45]. While final conclusions concerning the ancestral neural bodyplan of Annelida can therefore not yet be made, a comparison of the data currently available hints towards certain evolutionary trends which may have considerable bearing for our understanding of the origin of the polychaete (and annelid) nervous system.

Within Annelida *sensu lato*, it appears that a segmented body organization has been lost independently multiple times, but that it has been at least partly retained in the larval nervous system of some taxa that show no external signs of metamerism in the adult stage, such as echiurans [34,35,39] and sipunculans [47,51]. Similarly, multiple variations of the conventional rope-ladder-like nervous system have been described for various polychaetes. Accordingly, the number of connectives may range from one to five in adult polychaete annelids [44]. The presence of only one nerve in the setigerous abdomen of the adult and a reduced number of ventral commissures illustrates that the metameric arrangement of the nervous system is only weakly expressed in *Spirorbis *as well. These findings are consistent with an earlier description of another spirorbid polychaete, *Spirorbis moerchi *[52]. In this species, three pairs of ganglia and three ventral commissures, which are associated with the three larval segments, are formed, and these constitute the only neural components that hint towards a segmented nervous system [52]. Similar to the condition found in *S. spirorbis*, the ventral nerve cords are widely separated in the anterior body region before they fuse into one strand in the setigerous abdomen. Although the latter is externally segmented in the adult, this condition is not reflected in the nervous system [53-56]. The plasticity of annelid neural segmentation is also demonstrated by the variability concerning the neurogenesis pathways that may lead to the metameric arrangement of individual components of the segmented nervous system. As such, in the sabellid polychaete *Sabellaria alveolata*, two distinct modes of neural development are found, namely the simultaneous formation of the first three pairs of peripheral segmental neurons on the one hand and a strictly progressive formation in anterior-posterior direction of the ventral commissures on the other [45]. Taken together, these data indicate that the ontogenetic establishment of the segmented annelid nervous system is a highly dynamic process that must have undergone significant modifications from its ancestral developmental pathway in the various annelid lineages, and additional future studies on the subject are needed before we are able to fully understand the mechanisms that underlie annelid segmentation and nervous system evolution.

### Innervation patterns of the anterior appendages and the evolution of serpulid opercula

The serpulid operculum and its peduncle are commonly regarded as a modified tentacle (= radiolus) of the branchial crown [57-61]. Ever since Müller's [23] observation that the operculum forms at the tip of a pinnulate radiolus in individuals of the genus *Serpula*, the tentacles have been considered homologous to the operculum. This notion is still widely accepted, despite considerable phenotypic and developmental variation of serpulid opercula. As such, the operculum may either develop directly at the distal tip of a smooth peduncle without pinnules (e.g., in *Pomatoceros *and *Spirorbis*) or indirectly (e.g., in *Serpula*). In the latter case, the peduncle retains its pinnules only in some filogranin species. In addition, the Filograninae encompass a few non-operculate forms such as *Protula *(see Figure 1B).

Serpulid phylogeny has traditionally been founded to a large extent on these opercular characters [30]. For example, Pillai [25] proposed that the spirorbin opercula are not homologous to the ones of other serpulid species, and accordingly suggested a new classification of the spirorbin taxa, while ten Hove [62] proposed an evolutionary hypothesis for serpulid phylogeny based on a transformation series of the branchial crown alone. However, the latter author stressed at the same time the lack of other conclusive characters to substantiate serpulid interrelationships. To further assess this issue, neuronal innervation patterns of organ systems may be used as an independent test of homology *prior *to phylogenetic analysis, as illustrated by recent neuroanatomical studies on euchelicerates and pycnogonids (seaspiders) [63-65]. In addition, the comprehensive studies on the cephalic nervous system of polychaetes by Orrhage [7-9] highlight the relevance of such investigations for the homologization of anterior appendages in annelids.

The internal anatomy of the radioles has been analysed in several different serpulid species [60,66-68] including *Spirorbis *[53,68,69]. However, the present study on neurogenesis in *S. spirorbis *is the first one to raise serious doubts on the proposed homology of opercula and radioles in Spirorbinae based on differences concerning (i) the overall neural architecture, (ii) the innervation pattern, and (iii) the ontogenetic establishment of the nervous supply of the operculum and the radioles. In *Spirorbis*, the number of tentacles increases during development, resulting in an asymmetric branchial crown comprising five radioles on the right side and four on the left side in adult specimens [53,70]. In addition, classical studies have shown that development of the operculum is retarded compared to the radioles [71-73]. Indeed, the neuronal rudiments of the branchial crown are formed prior to the ones of the operculum. However, the differentiation of the opercular neurons advances more rapidly during subsequent development, with the opercular nerve lining the entire peduncle even before the radiole nerves differentiate (Figure 6B). In addition, exactly the same number of radioles and pinnules arises on both sides (Figure 6C). Accordingly, we regard the asymmetric arrangement in the adult a secondary condition, probably constrained by limited space in the cephalic region rather than being the result of evolutionary transformation events of a radiole into an operculum.

The general neural architecture of *Spirorbis *shows that each radiole comprises three nerves [[68], present study]. A similar condition has been described for *Pomatoceros*, where two nerves are situated in the abfrontal corners ("external branchial nerves") and a third one frontally in close proximity to the food groove ("internal branchial nerve"; [60,68]). Thereby, the abfrontal nerves lie outside the basement membrane of the epidermis, whereas the frontal nerve lies just inside the basement membrane. All three branchial nerves send branches into the pinnules. This tripartite arrangement has been documented for *Pomatoceros *not only for the radioles and pinnules, but also for the peduncle [68]. At the top of the peduncle the internal and external peduncular nerves enter the operculum through a small, single gap. There, all three nerves furcate, thus giving rise to numerous small branches which can be traced to the rim surrounding the distal pole of the operculum [68]. This tripartite arrangement in both the radioles and the operculum is in accordance with the hypothesis of Müller [23]. Hanson [68] described three branchial nerves also for other serpulid species such as *Vermiliopsis*, *Protula*, *Salmacina *and *Hydroides*, but he did not mention explicitly the neuronal innervation of the peduncle. Only for *Hydroides *he clearly stated that the internal and external peduncular nerves are similar to those of the filaments of *Pomatoceros*. However, in juvenile *S. spirorbis*, there are no three parallel nerves present in the peduncle. Instead, a single nerve loop lines the margin of the opercular stalk. Furthermore, the two strands of this nerve loop do not lie close together at the transition between the peduncle and the operculum. Accordingly, the number, spatial distribution, and site of emergence of the opercular nerves in *Spirorbis *are unique and do not correspond to the situation found in the radioles and in the opercular peduncle of *Pomatoceros *and *Hydroides*.

Probably the most striking argument against a shared evolutionary ancestry of the radioles and the operculum in *Spirorbis *is revealed when the cerebral innervation sites of both structures are compared. While the opercular nerves appear to emerge from the dorsal commissures of the cerebral ganglion, the two main nerve bundles of the radioles are associated with the ventral part of the cerebral ganglion. The nerves of the radioles show no connection to the opercular nerves. The innervation of anterior appendages in the Serpulidae has been already investigated in detail for selected species, albeit not in *Spirorbis*, and used for homology assessments [8]. Based on his findings, Orrhage described ten different nerve roots of the branchial crown that partly originate from the ventral and partly from the dorsal commissures on either side of the cerebrum, before finally forming one anterior branchial crown nerve. It might be that one or more of these nerve roots have gained new functions in spirorbin taxa that brood in their operculum [22]. The resolution of the data presented herein does not allow for unambiguous distinction of individual nerve roots. The question whether this is due to limitations of the used immunolabeling techniques or a lack of differentiation in early developmental stages has to be left open. Orrhage [8] does not explicitly describe the innervation of the operculum in *Spirorbis*. Nethertheless, we expect that the neuronal root of the operculum would be merged with the other nerve roots to form a single branchial crown nerve in adults, in case that the operculum represents indeed a modified radiole.

The most recent phylogenetic studies propose that *Hydroides *belongs to the *Serpula-*clade, the sister group of the *Pomatoceros*-clade (Figure 1B). This implies that the operculum with three peduncular nerves, that evolved from a radiole of the branchial crown, is most likely a shared character of these two sister clades. So far, nothing is known about the neuronal innervation of the operculum in species of the *Protula*-clade, where some of its members do not even have an operculum. Therefore, any final conclusions about the ancestral condition of the operculum at the base of the serpulid tree are premature. In principal, several scenarios appear possible in the light of the current phylogenetic hypothesis (Figure 1B): 1) opercula evolved at least twice independently, once in the *Serpula*/*Pomatoceros*-clade and once in the *Protula*/*Spirorbinae*-clade, 2) the operculum was lost at the base of the Spirorbinae, and secondarily evolved again within this group as an independent opercular structure, or (3) the operculum is a shared ancestral feature of the Serpulidae that was - together with its neural innervation pattern - secondarily modified in the descending lineages. Based on our data on the neural innervation pattern of the operculum in *Spirorbis *we favour the former alternative, although additional comparative data on the developmental programme (e.g., gene expression analysis) that underlies radiole and operculum formation in the respective serpulid clades are needed to finally settle this issue.

## Conclusion

Our data suggest that the operculum in *Spirorbis *is not a derivative of the branchial crown but an independent structure which originates from the bodywall around the mouth. These findings corroborate a recent molecular phylogenetic study that suggests convergent evolution of direct operculum development, once at the base of the *Pomatoceros*-group and once along the line leading to the Spirorbinae [24]. In addition, the new data on radiole and operculum innervation presented herein strongly argue for the following scenario:

1. The operculum of *Spirorbis *is a prostomial appendage and does not belong to the branchial crown. It is thus not homologous to the radioles.

2. The operculum of *Spirorbis *is not homologous to the opercula of *Serpula, Hydroides *and *Pomatoceros*. In the latter three, the operculum is indeed likely to have a shared evolutionary origin with the radioles. Therefore, the opercula of *Serpula, Hydroides *and *Pomatoceros *are considered homologous to each other.

Based on our data we predict that future neuroanatomical and developmental studies of the branchial crown of selected species, especially of the *Protula*-clade (including the former Filograninae), will further contribute to our understanding of both the evolution of opercular structures in serpulid polychaetes and will also aid in our quest for the eventual placement of this group within the annelid phylogenetic tree.

## Methods

### Animals

Adult specimens of *Spirorbis *cf. *spirorbis *were collected in the intertidal around Roscoff, France, in June 2006 and 2007, respectively. The calcareous tubes were crushed with tweezers under a stereo microscope in order to obtain the egg strings which contained embryos of various developmental stages (Figure 1A). Developmental stages (encapsulated embryos, free-swimming larvae, settled larvae, and juveniles) were cultured in embryo dishes at 17-19°C in Millipore-filtered seawater (MFSW). Prior to fixation, the specimens were anesthetized in a 1:1 dilution of MFSW and MgCl_2 _(7%). They were then fixed at room temperature in 4% paraformaldehyde in 0.1 M phosphate buffer (PB) for 1.5-3 h or overnight at 4°C, washed three times in PB, and stored at 4°C in PB containing 0.1% NaN_3_. Tubed juveniles were decalcified in 50 mM EGTA and washed thrice in PB prior to storage at 4°C.

### Scanning electron microscopy (SEM)

Stored animals were washed in distilled water, postfixed in 1% osmium tetroxide, washed twice in distilled water, and dehydrated in a graded alcohol series. After critical point drying in acetone, the samples were mounted on SEM stubs, sputter-coated with gold-palladium, and analyzed with a JEOL JSM-6335-F SEM (JEOL, Tokyo, Japan).

### Immunolabeling

The following steps were all performed at 4°C. Antibody staining was preceded by tissue permeabilization for 1 h in 0.1 M PB with 0.1% NaN_3 _and 0.1% Triton X-100 (PTA), followed by incubation in block-PTA [6% normal goat serum (Sigma-Aldrich, St. Louis, MO, USA) in PTA] overnight. The primary antibodies, polyclonal rabbit anti-serotonin (Zymed, San Francisco, CA, USA, dilution 1:800), polyclonal rabbit anti-FMRFamide (Chemicon, Temecula, CA, USA, dilution 1:400), and monoclonal mouse anti-acetylated α-tubulin (Sigma-Aldrich, dilution 1:1000), all in block-PTA, were either applied separately or in a mixed cocktail for 24 h. Subsequently, specimens were rinsed in block-PTA with three changes over 6 h and incubated thereafter with 4'6-diamidino-2-phenyl-indole [DAPI (Invitrogen, Eugene, OR, USA)] and secondary fluorochrome-conjugated antibodies [goat anti-rabbit FITC (Sigma-Aldrich), dilution 1:400; goat anti-rabbit Alexa Fluor 594 (Invitrogen), dilution 1:1000; goat anti-mouse FITC (Sigma-Aldrich), dilution 1:400] in block-PTA for 12-20 h. Finally, the specimens were washed three times in PB without NaN_3_, once in distilled water, and thereafter dehydrated through a graded ethanol series (30%, 50%, 70%, 80%, 90%, 3 × 100%), cleared with benzyl benzoate:benzyl alcohol (2:1), and mounted on glass slides.

### Confocal laserscanning microscopy and 3D reconstruction

Immunolabeled specimens were analyzed for each antibody separately for a minimum of 10 individuals per developmental stage. Altogether, about 60 image stacks of optical sections were recorded as Z-wide-projections with 0.1-0.5 μm step size using a Leica DM IRE2 fluorescence microscope equipped with a Leica TCS SP 2 confocal laserscanning unit (Leica, Wetzlar, Germany). The Z-stacks were projected into maximum intensity pixel as well as depth-coded images. In addition, corresponding light micrographs were recorded for most specimens. The three-dimensional computer reconstructions were generated with the imaging software Imaris v. 5.5.3 (Bitplane, Zürich, Switzerland) using surface rendering algorithms. Image adjustment was done with Adobe Photoshop CS2 and arrangements of plates with Adobe Illustrator CS3 (Adobe Systems, San Jose, CA, USA).

## Authors' contributions

NB performed research, analyzed data, and drafted the manuscript. AW designed and coordinated research and contributed to data analysis and writing of the manuscript. Both authors conceived the study and read and approved the final version of the manuscript.
